# Cross-cultural adaptation and reliability of the European Portuguese version of the Musculoskeletal Health Questionnaire: A methodological study

**DOI:** 10.1371/journal.pone.0308623

**Published:** 2024-08-08

**Authors:** Hermínia Ribeiro, Eduardo Brazete Cruz, Ana Castro, Ana Rodrigues, Bruno Heleno, Teresa L. Dias, Diogo Pires

**Affiliations:** 1 Instituto Politécnico de Setúbal, Escola Superior de Saúde, Setúbal, Portugal; 2 Comprehensive Health Research Centre, NOVA Medical School, Universidade Nova de Lisboa, Lisboa, Portugal; 3 Centro de Linguística da Universidade NOVA de Lisboa (CLUNL), Lisboa, Portugal; 4 EpiDoC Unit, NOVA Medical School, NOVA University of Lisbon, Lisbon, Portugal; 5 Unidade de Reumatologia, Hospital dos Lusíadas, Lisbon, Portugal; 6 NOVA Medical School, NOVA University of Lisbon, Lisbon, Portugal; UFSCar: Universidade Federal de Sao Carlos, BRAZIL

## Abstract

**Purpose:**

To culturally adapt the Musculoskeletal Health Questionnaire (MSK-HQ) to European Portuguese and evaluate its reliability in individuals with musculoskeletal conditions.

**Materials and methods:**

The study was carried out in two phases. In the first phase, the MSK-HQ was translated and culturally adapted. In the second phase, a longitudinal observational study was carried out with a convenience sample of participants with musculoskeletal conditions. Data collection began at the start of physiotherapy treatments by filling in the MSK-HQ and Numeric Pain Rating Scale (NPRS). After 4–7 days, the participants were asked to fill out the MSK-HQ once again, as well as the Patient Global Improvement Change (PGIC) scale. The data collected was used to study internal consistency, test-retest reliability, and measurement error. Floor and ceiling effects were also analysed.

**Results:**

The MSK-HQ was successfully translated and adapted into European Portuguese. The second phase of the study had a sample of 191 participants. This study demonstrated high internal consistency (Cronbach’s α = 0.885) and excellent test-retest reliability (ICC_(2,1)_ = 0.908). The analysis of measurement error resulted in an SEM of 2.818 and an SDC at 7.811. No floor or ceiling effect was observed.

**Conclusions:**

The MSK-HQ-PT is a reliable instrument for measuring musculoskeletal health. Further studies on its validity and responsiveness are needed.

## Introduction

Musculoskeletal (MSK) conditions have a high prevalence in the global population and are a major cause of years lived with disability [1]. MSK conditions, such as low back pain, neck pain, or osteoarthritis, are among the health conditions with the greatest associated individual and social impact [2]. While pain and disability primarily affect the physical component of health, the decline in mental and social well-being among individuals with MSK conditions has also been well described in the literature [3, 4]. These three health components share a complex and reciprocal relationship, as evidenced by the low levels of health-related quality of life experienced and reported by individuals with MSK conditions [5, 6]. As a result, they pose a substantial economic impact on national economies, leading to significant social and healthcare expenditures, along with numerous other indirect costs [3, 4].

In the past decade, health literature and policy have focused on raising awareness about the societal impact and costs associated with MSK conditions, empowering individuals, and promoting prevention and cost-effective treatments [7]. However, it is currently recognized that musculoskeletal health still is not a priority in healthcare, which translates into a misalignment with the best current recommendations [8, 9]. It is worth highlighting the need to move towards person-centred care, which includes effective communication, the use of shared decision-making processes or the use of valid and reliable outcome measures (among other components) [10]. Considering this perspective, patient-reported outcome measures (PROMs) have gained significant prominence. They enhance communication, facilitate discussions between patients and healthcare professionals, foster shared decision-making, and capture essential health domains (and problems) that might otherwise remain unmeasured (such as depression or self-efficacy) [11–13].

However, the regular utilization of PROMs in individuals with MSK conditions has encountered challenges. On one hand, generic PROMs like the EQ-5D serve the purpose of comparing MSK conditions, but they seem to exhibit limited responsiveness and fail to encompass the most pertinent constructs related to musculoskeletal health [14–16]. On the other hand, the use of construct or condition-specific PROMs (e.g., Pain Self-Efficacy Questionnaire or Knee Injury and Osteoarthritis Outcome Score) in a real-world practice context can be difficult due to the number of relevant constructs to measure, the length of the available PROMs and the associated time burden for patients and clinicians [12, 17, 18]. Moreover, the growing consensus regarding common characteristics and prognostic factors across MSK conditions implies a need for a comprehensive response and cross-cutting assessment by healthcare providers [10, 19, 20].

To address these issues, the Musculoskeletal Health Questionnaire (MSK-HQ) was developed [21]. Although the MSK-HQ is a generic PROMs, it captures the most important domains of musculoskeletal health for patients and health professionals through 14 items with 5 response options [12]. The final score is obtained by the sum of the responses ranging from 0 to 56 (better musculoskeletal health). Recent validation studies for different languages and cultures showed that MSK-HQ is a valid and reliable instrument for use in a wide range of MSK conditions [12, 21, 22–25]. To expand the use of MSK-HQ to Portugal, the translation and analysis of its psychometric properties are essential. Therefore, the aim of this study was to culturally adapt the Musculoskeletal Health Questionnaire to European Portuguese and evaluate its reliability in individuals with musculoskeletal conditions.

## Materials and methods

This methodological study was carried out in two phases: the first consisted of the translation and cross-cultural adaptation of the original version of the MSK-HQ into European Portuguese; the second focused on the analysis of the reliability (internal consistency, test-retest reliability and measurement error) of the Portuguese version of the MSK-HQ in individuals with MSK conditions. Floor and ceiling effects were also analysed. Phase 2 of this study was designed according to the recommendations and definitions of COSMIN [26].

This study was submitted and approved by the Specialised Research Ethics Committee of the Instituto Politécnico de Setúbal, Portugal (number 65A/HC/2021). All participants provided their written informed consent after receiving information about the study.

### Translation and cross-cultural adaptation process (phase 1)

Before starting the translation and cross-cultural adaptation process, authorization was requested from the original authors of the MSK-HQ. This process was carried out according to the following recommendations proposed by Beaton et al. (2000) [27], as follows:

- Translations: two translators, bilingual European Portuguese native speakers, produced independent translations from the original version of MSK-HQ into European Portuguese.
- Synthesis of the translations: A single version was produced after consensus among the two translators and the research team.
- Back translation: two other translators, native English-speakers and blind to the original of MSK-HQ, back-translated the synthesized version of the MSK-HQ into the original English language.
- Expert committee review: An expert committee composed by one methodologist, one linguistic expert and five health professionals (one rheumatologist, two general practitioners and two physiotherapists) was formed. The role of the expert committee was to analyse all translations, reach consensus and propose a pre-final version for field testing.
- Test of the Prefinal Version: The pre-final version was cognitively debriefed with a sample of 20 native Portuguese patients with musculoskeletal pain and heterogeneous clinical and sociodemographic characteristics [28]. Patients were interviewed by two researchers to assess the comprehensibility and acceptancy of the MSK-HQ pre-final version. Completion time, doubts and suggestions about the MSK-HQ were recorded.
- Appraisal of the Adaptation Process: a final audit of the whole process was carried out by the research team together with the original authors, who also addressed the necessary changes based on the participants’ feedback to produce the final version of MSK-HQ-PT.

### Reliability (phase 2)

#### Recruitment and participants

An independent sample of patients with musculoskeletal pain was used to investigate the reliability of the Portuguese version of the MSK-HQ. A longitudinal design study with a follow-up of 4 to 7 days was conducted between March 2021 and December 2021. Consecutive patients with musculoskeletal pain were recruited from the waiting list of 7 outpatient clinics from 5 different regions in Portugal. Local physiotherapists identified potential participants following a standardized recruitment protocol.

Participants were considered eligible if they had non-specific musculoskeletal pain, age equal to or greater than 18 years, were able to read and write European Portuguese, and were starting a physiotherapy intervention. They were excluded from the study if they had signs and symptoms compatible with a specific pathology such as neoplastic (visceral/malignant pain), systemic (e.g., rheumatoid arthritis, lupus erythematosus, vasculitis, etc.), infectious (presence of a fever or rheumatism), visceral, neurological, circulatory, or any other red-flag (e.g., signs and symptoms of radicular compression, cauda equina, fracture/fracture risk associated with osteoporosis, among others); or had any other contraindication to physiotherapy; or had undergone surgery in the last 6 months; or had undergone conservative intervention in the last 3 months.

At baseline, all eligible participants who agreed to participate in the study completed a questionnaire booklet containing sociodemographic and clinical data, the MSK-HQ-PT and the Numeric Pain Rating Scale (NPRS). Then, 4 to 7 days after the first assessment, participants completed the MSK-HQ-PT and the Patient Global Improvement Change (PGIC). Given the construct being analyzed (musculoskeletal health) and the questionnaire’s items and response options, this time interval was chosen to ensure participant stability and avoid recall bias. All instruments were completed on paper by the participants. Local physiotherapists ensured the instruments were filled out in a calm environment under consistent conditions for both time points, without interfering in participants’ completion or responses.

#### Sample size

Following current recommendations, a minimum sample of 50 participants for test-retest reliability analysis and 100 participants for internal consistency analysis were defined [29]. Assuming that larger samples are preferable, the recruitment process continued for the planned duration of the study.

#### Study instruments

The MSK-HQ consists of 14 items to capture relevant musculoskeletal health domains prioritised by patients and clinicians, including pain severity, physical function, work interference, social interference, sleep, fatigue, emotional health, physical activity, independence, understanding, confidence to self-manage and overall impact). Patients rate how much their musculoskeletal condition has affected each of the domains in the previous two weeks using a 5-point scale, from “not at all” (4 points) to “extremely” (0 points) [12]. The final score is obtained by the sum of the responses ranging from 0 to 56 (better musculoskeletal health). This score measures overall musculoskeletal health status using a formative model [12]. A fifteenth item assessing physical activity is not included in final score. The original version of the MSK-HQ showed excellent test-retest reliability (ICC = 0.84), adequate internal consistency as one scale (Cronbach’s α = 0.88) and strong convergent validity (correlations 0.81–0.88) [12].

The NPRS is a single item instrument for measuring pain intensity that has been widely used and validated in multiple types of conditions and adults/users [30–33]. The scale has 11 points and requires the respondent to select a whole number between 0 (no pain) and 10 (maximum pain) that best reflects the intensity of pain at that moment (“Please select the number that best represents the intensity of pain you currently feel in the area where you have your musculoskeletal problem.”) [31, 34, 35]. The PGIC is a single item, self-report scale that assesses the individuals’ perceptions of improvement, reflecting not only the magnitude of changes in outcomes, but also the personal significance of these changes [36]. Respondents rate their improvement on a 7-item scale to the following question: “Please indicate the degree of change (if any) in your overall activity limitations, symptoms, emotions, and quality of life since beginning treatment at this institution in relation to your pain” [37]. This tool provides important easily interpretable information and is therefore widely used both scientifically and clinically to assess global changes in health status [36, 38]. The PGIC has been cross-culturally validated to the European Portuguese language and showed adequate psychometric properties [36, 39].

### Statistical analysis

All data analysis was performed using the IBM SPSS Statistics ^®^ software, version 27 for Windows 10 Home^®^. A p-value <0.05 was considered to indicate statistical significance. Descriptive statistics including means (SD) and frequencies (%) were used to summarize participants’ characteristics.

Missing item data were counted and imputed following recommendations of previous studies analysing MSK-HQ psychometric properties [21]. If there are 3 or fewer missing items, the score should be averaged, and the value assigned accordingly. If the number of missing items was greater than 3, the data were eliminated.

Internal consistency is defined as the relationship between items, which in turn depends on the unidimensionality of the scale, i.e., the homogeneity of the items [26]. Using the baseline data of MSK-HQ-PT, this psychometric property was analysed using Cronbach’s alpha, whose value varies between 0 and 1 [26, 40]. The MSK-HQ-PT was considered to have adequate internal consistency if a Cronbach alpha ≥0.70 and <0.90 was found [41]. In addition, the internal consistency analysis was complemented using item-total correlations and inter-item correlations, which made it possible to check whether these correlations were strong and verified by all items.

Test-retest reliability is the proportion of the total variance in the measures that correspond to “true” differences between users [26]. It was calculated from the paired MSK-HQ-PT total scores obtained at baseline and after 4–7 days. Only “clinically stable” participants were included in this analysis, i.e., those who did not perceive any improvement in their condition over the defined period. The PGIC was used to identify these participants. This means that only data from individuals who had answered items 1 (“No change (or condition worsened)”), 2 (“Almost the same, with no visible change”) or 3 (“Slightly better, but no considerable changes”) of PGIC were used. For this analysis, the Intraclass Correlation Coefficient (ICC_2,1_) was calculated. An ICC_2,1_ value ≥ 0.70 was considered acceptable [12, 26]. In addition, the test-retest reliability of each item was analysed individually using the Kappa coefficient of agreement, more specifically through the weighted kappa, using quadratic functions [29].

Measurement error is defined as the systematic and random error in an individual’s score that is not attributable to true change in the construct being measured [26]. Measurement error was estimated using the standard error of measurement (SEM) and the smallest detectable change (SDC) [29]. The formula SEM = standard deviation ×√1−*R* where R corresponds to the reliability of the instrument (using ICC_2,1_) was used [42, 43]. The SDC was calculated using the following mathematical formula: SDC = 1.96×√2×SEM, where 1.96 is the z value of the 95th percentile of a distribution [29].

The “floor” and “ceiling” effects was calculated using the value obtained from the SDC, i.e., the lower limit was equal to or less than the SDC and the upper limit was equal to 56 (maximum MSK- HQ) minus the SDC. The “floor” and “ceiling” effects were present if more than 15% of the participants achieved the lowest possible score (“floor” effect) or the highest possible score (“ceiling” effect) [29].

## Results

### Cross-cultural adaptation process

The translation process was performed without any major difficulties. During this process, the expert committee introduced a few minor adaptations to words and expressions that are not commonly understood by Portuguese speakers, aiming to enhance comprehensibility. For example, the literal translations of "aches", "fatigue" or "jobs around the house" are not commonly used and have been replaced by synonyms closer to the everyday language of European Portuguese speakers.

Of the 20 patients in the test of the prefinal version of the MSK-HQ, 14 (70%) were women and 6 (30%) were men. Their mean age was 48.8 years. Sixteen patients were active workers and two reported being unable to work due to musculoskeletal pain. Most of the patients had musculoskeletal pain for more than 3 months and took medication for pain. The pain location varied, but the shoulder (5 participants) and the knee (4 participants) were the most mentioned symptomatic areas. The mean current pain intensity was 7.1 points on the NPRS, and the average musculoskeletal health score measured by the MSK-HQ-PT was 32.8. The interviews conducted with 20 patients did not yield significant changes to the wording of the MSK-HQ-PT. In general, all participants considered the questionnaire, including its instructions, to be simple, clear, and understandable. However, it is important to highlight those 7 participants identified potential comprehension issues with items 12 and 13 due to the provided examples in brackets. The research team unanimously agreed that removing these examples would enhance the simplicity and comprehensibility of the items. The final version of the MSK-HQ-PT incorporated these revisions (S1 Appendix). The average time taken to complete the MSK-HQ-PT was 5 minutes and 5 seconds.

### Reliability

A total of 191 participants completed the questionnaire booklet at baseline. Table 1 presents baseline sociodemographic and clinical data of the sample. Of these, 135 completed the second assessment (4 to 7 days later). Fifty-six of the 135 participants were considered “stable” and therefore were included in the test-retest analysis. Data from 191 participants were considered for internal consistency analysis. No floor or ceiling effect was observed.

**Table 1:** Characteristics of the participants and summary measures of the questionnaires.

Variable	N	Mean (SD) or N (%)
**Age (years)**	190	50,9
**Gender**		
Female	125	65,8
Male	65	34,2
**Weight (kg)**	190	74,3 (13,7)
**Height (m)**	188	1,7 (0,1)
**BMI (kg/m2)**	188	26,9 (4,4)
**Absence from work due to your condition in the last year**		
Yes	57	32,2
No	120	67,8
**Duration of symptoms**		
0–3 months	54	28,7
3–6 months	29	15,4
6–12 months	31	16,5
More than 12 months	74	39,4
**Pain localisation**		
Back	41	21,6
Neck	33	17,4
Knee	47	24,7
Shoulder	65	34,2
Others	4	2,1
**Pain relief medication**		
Yes	87	46
No	102	54
**MSK-HQ-PT**	190	31,8 (9,5)
**Pain Intensity (NPRS)**	187	6,2 (2,3)

At baseline, 174 participants (91.1%) filled out the MSK-HQ-PT completely. Fourteen questionnaires had 1 missing item responses, 2 had 2 missing items responses and 1 had more than 3 missing items responses. Items 12 and 13 had the most missing responses (2,6%).

The MSK-HQ-PT has adequate internal consistency, with a Cronbach’s α of 0.885. In relation to inter-item correlations, positive correlations ranged from 0,053 (between items 3 and 12) to 0,70 (between items 10 and 11). Regarding item-total score correlations, they ranged from 0.235 to 0.702. The elimination of any item did not significantly alter the Cronbach’s α coefficient, i.e., there was no item whose exclusion substantially increased the internal consistency of the MSK-HQ-PT.

Based on total score, the ICC_2,1_ of MSK-HQ-PT was 0.908 (95% CI 0.825–0.949), indicating excellent test-retest reliability. The Kappa coefficient of agreement for the MSK-HQ-PT items ranged from 0.434 (95%CI 0.207–0.661) for item 7 to 0,798 (95%CI 0.692–0.903) for item 9 (Table 2). The analysis of measurement error resulted in an SEM of 2,818 (95% CI 2,098–3,88) and an SDC at 7,811 (95% CI 8,815-10-774).

**Table 2:** Item-item test-retest reliability using squared weighted Kappa coefficients in the MSK-HQ-PT.

Item	Kappa	CI (95%)
**1**	0.500	0.246–0.754
**2**	0.749	0.636–0.862
**3**	0.749	0.626–0.872
**4**	0.641	0.499–0.782
**5**	0.787	0.691–0.883
**6**	0.563	0.273–0.854
**7**	0.434	0.207–0.66
**8**	0.603	0.374–0.832
**9**	0.798	0.692–0.903
**10**	0.748	0.610–0.886
**11**	0.724	0.569–0.878
**12**	0.653	0.479–0.826
**13**	0.488	0.253–0.723
**14**	0.613	0.468–0.757

## Discussion

The aims of this study were to cross-culturally adapt the MSK-HQ into European Portuguese and to examine its reliability. The MSK-HQ was translated and cross-culturally adapted into European Portuguese successfully, according to international guidelines [27] and showed excellent reliability (internal consistency and test-retest reliability). Although other fundamental psychometric properties need to be analysed, these results support the potential use of MSK-HQ-PT in clinical and research contexts, with the aim of measuring musculoskeletal health in patients with musculoskeletal pain conditions.

In general, the MSK-HQ-PT was considered clear, relevant, and understandable. However, some issues were reported regarding items 12 (Understanding of your condition and current treatment) and 13 (Confidence in being able to manage your symptoms), which were addressed by modifying the wording of those items. Similar difficulties were reported during the process of translation and cross-cultural adaptation of the German and Danish versions [25, 44]. Current evidence has supported the importance of these domains in the context of musculoskeletal pain, as well as their importance to patients and clinicians [12, 45]. The fact that these domains may not be intuitive or familiar to patients, and are difficult to operationalize in a single question, may help explain these issues.

The MSK-HQ-PT demonstrated high internal consistency with similar values of Cronbach’s alpha (0.885) to the original English version (0.88) [12], Italian version (0.87) [46], Arabic version (0.88) [47], and Norwegian version (0.86) [24]. Therefore, the various items of the MSK-HQ-PT seemed to have high interrelatedness. Looking at the analysis of item-item and item-total correlations, it is relevant to highlight two findings. First, items 10 and 11 showed higher correlation values (0.7) than recommended (0.2 to 0.5), revealing a possible domain overlapping [29]. The lack of previous studies carrying out this type of analysis prevents a consistent interpretation of this data. Second, item 12 has lower (0.235) than desirable correlation values with the total score (> 0.3) [43], although there would be no significant increase in Cronbach’s alpha if this item were removed (increase of 0.006). Identical results were reported for this item in the English original and Italian versions [12, 46], which may justify a future analysis of the relevance of this item/domain.

The test–retest reliability of the MSK-HQ-PT was found to be excellent for the total score (ICC = 0.908), in line with similar studies that demonstrated adequate test–retest reliability, with ICC values ranging from 0.83 to 0.95 [12, 24, 25, 46, 47]. Similarly, all individual items showed adequate test-retest reliability (Kappa coefficient ranged from 0.434 to 0.798), in line with findings found in the German version [25] and the recommended values [48]. Furthermore, this study analysed the measurement error (SEM and SDC) resulting from repeated measures. A SEM of 2.818 (1,6% of the maximum score) and an SDC of 7.811 (4,4% of the maximum score) were calculated, representing low values considering the full range of the scores from 0–56. Both values are in line with those reported in previous studies that conducted similar analyses [24, 25, 46, 47]. The implication of a low measurement error is that the MSK-HQ-PT may be an appropriate outcome measurement to assess change in musculoskeletal health during an intervention in patients with musculoskeletal pain. Nevertheless, the ability of the MSK-HQ-PT to detect changes in musculoskeletal health over time (responsiveness) should be analyzed in future studies.

The results of this study should be interpreted in the light of some limitations. One possible limitation is the choice of a second measurement (4 to 7 days) for the test-retest reliability analysis. This short interval time reduces the chances of a change in the measured construct but increases the possibility of recall bias. Two reasons were behind this choice: 1) musculoskeletal health is a complex construct that can be influenced by multiple contextual and health-related aspects of patients, therefore, shorter intervals time to repeat measurement are preferable; 2) the MSK-HQ consists of 14 items, which reduces the possibility of recall bias in a measurement after 4 to 7 days. Another potential limitation is that the study used a convenient sample which might influence the degree of generalization of the findings. The strengths of this study include the adequate sample size and the comprehensive analysis of the parameters that contribute to the reliability of PROMs.

Given the results of this study, the MSK-HQ-PT emerges as a relevant tool for both clinical practice and research involving patients with musculoskeletal pain. However, it is important to note that this study constitutes just the initial phase of psychometric property analysis for the MSK-HQ-PT. Future studies focusing on validity and responsiveness analysis are needed.

## Conclusions

The MSK-HQ-PT is a reliable instrument for measuring musculoskeletal health. Further studies on its validity and responsiveness are needed.

## Supporting information

S1 Appendix Final version of the MSK-HQ-PT. (DOCX)

S2 Appendix Original version of the MSK-HQ-UK. (DOCX)

S3 Appendix Data set used in analysis. (XLSX)
