# Supplementary material for: Cross-cultural adaptation and reliability of the European Portuguese version of the Musculoskeletal Health Questionnaire: A methodological study
Source: PLoS One. 2024 Aug 8;19(8):e0308623. doi: 10.1371/journal.pone.0308623 (PMC11309473; doi:10.1371/journal.pone.0308623)
Supplement: S1 Appendix — (DOCX) [file pone.0308623.s001.docx]

## MUSCULOSKELETAL HEALTH QUESTIONNAIRE (MSK-HQ)

VERSÃO EM PORTUGUÊS EUROPEU

Este questionário é sobre os seus sintomas tais como desconforto, dor e/ou rigidez nas suas **articulações, costas, pescoço, ossos e músculos.** Por favor, concentre-se no(s) problema(s) de saúde que o/a levou/ levaram a procurar tratamento neste serviço.

*Para cada questão,* ***assinale (√) a opção*** *correspondente à afirmação*

*que melhor o/a descreve* ***nas últimas 2 semanas***

| 1. Dor/rigidez durante o dia  Nas últimas 2 semanas, no geral, quão intensas foram as suas dores e/ou rigidez articular ou muscular ao longo do dia? | Nada | Ligeiramente | Moderadamente | Muito | Muitíssimo |
| --- | --- | --- | --- | --- | --- |
|  | □ | □ | □ | □ | □ |
| 2. Dor/rigidez durante a noite  Nas últimas 2 semanas, no geral, quão intensas foram as suas dores e/ou rigidez articular ou muscular durante a noite? | Nada | Ligeiramente | Moderadamente | Muito | Muitíssimo |
|  | □ | □ | □ | □ | □ |
| 3. Andar  Nas últimas 2 semanas, de que forma os seus sintomas interferiram na sua capacidade de andar? | Nada | Ligeiramente | Moderadamente | Muito | Incapaz de andar |
|  | □ | □ | □ | □ | □ |
| 4. Lavar/Vestir  Nas últimas 2 semanas, de que forma os seus sintomas interferiram na sua capacidade de se lavar ou vestir? | Nada | Ligeiramente | Moderadamente | Muito | Incapaz de me lavar ou vestir sozinho/a |
|  | □ | □ | □ | □ | □ |
| 5. Níveis de atividade física  Nas últimas 2 semanas, quão difícil tem sido fazer atividade física no nível que deseja (p. ex., caminhar ou correr) devido aos seus sintomas articulares ou musculares? | Nada | Ligeiramente | Moderadamente | Muito | Incapaz de fazer atividade física |
|  | □ | □ | □ | □ | □ |
| 6. Trabalho/rotina diária  Nas últimas 2 semanas, de que forma os seus sintomas articulares ou musculares interferiram no seu trabalho ou na sua rotina diária (incluindo tarefas e trabalhos domésticos)? | Nada | Ligeiramente | Moderadamente | Muito | Muitíssimo |
|  | □ | □ | □ | □ | □ |
| 7. Atividades sociais e de lazer (*hobbies*)  Nas últimas 2 semanas, de que forma os seus sintomas articulares ou musculares interferiram nas suas atividades sociais e de lazer? | Nada | Ligeiramente | Moderadamente | Muito | Muitíssimo |
|  | □ | □ | □ | □ | □ |
| Por favor vire a página e continue | | | | | |

| 8. Necessidade de ajuda  Nas últimas 2 semanas, com que frequência precisou de ajuda de outras pessoas (incluindo família, amigos ou cuidadores) devido aos seus sintomas articulares ou musculares? | Nunca | Raramente | Algumas vezes | Frequentemente | Sempre |
| --- | --- | --- | --- | --- | --- |
|  | □ | □ | □ | □ | □ |
| 9. Sono  Nas últimas 2 semanas, com que frequência teve problemas em adormecer ou manter-se a dormir devido aos seus sintomas articulares ou musculares? | Nunca | Raramente | Algumas vezes | Frequentemente | Todas as noites |
|  | □ | □ | □ | □ | □ |
| 10. Cansaço ou pouca energia  Nas últimas 2 semanas, quão cansado ou com pouca energia se sentiu? | Nada | Ligeiramente | Moderadamente | Muito | Muitíssimo |
|  | □ | □ | □ | □ | □ |
| 11. Estado emocional  Nas últimas 2 semanas, quão ansioso/a ou desanimado/a se sentiu devido aos seus sintomas articulares ou musculares? | Nada | Ligeiramente | Moderadamente | Muito | Muitíssimo |
|  | □ | □ | □ | □ | □ |
| 12. Compreensão do seu problema e tratamento atual  Refletindo sobre os seus sintomas articulares e musculares, quão bem sente que compreende o seu problema e o tratamento atual? | Completamente | Muito | Moderadamente | Ligeiramente | Nada |
|  | □ | □ | □ | □ | □ |
| 13. Confiança na capacidade de gerir os seus sintomas.  Nas últimas 2 semanas, quão confiante se sentiu para gerir sozinho/a os seus sintomas articulares ou musculares? | Muitíssimo | Muito | Moderadamente | Ligeiramente | Nada |
|  | □ | □ | □ | □ | □ |
| 14. Impacto geral  Nas últimas 2 semanas, de que forma é que os seus sintomas articulares ou musculares o/a incomodaram? | Nada | Ligeiramente | Moderadamente | Muito | Muitíssimo |
|  | □ | □ | □ | □ | □ |

| **15. Níveis de atividade física**  Na última semana, quantos dias fez pelo menos 30 minutos de atividade física com intensidade suficiente para aumentar o batimento do coração? Este tipo de atividade pode incluir desporto, exercício, caminhadas rápidas ou a utilização da bicicleta em lazer ou para deslocações, mas não deve incluir as tarefas domésticas ou a atividade física realizada no seu trabalho. | | | | | | | |
| --- | --- | --- | --- | --- | --- | --- | --- |
| Nenhum  □ | 1 dia  □ | 2 dias  □ | 3 dias  □ | 4 dias  □ | 5 dias  □ | 6 dias  □ | 7 dias  □ |

Obrigado por preencher este questionário.
