# Supplementary material for: Cross-cultural adaptation and reliability of the European Portuguese version of the Musculoskeletal Health Questionnaire: A methodological study
Source: PLoS One. 2024 Aug 8;19(8):e0308623. doi: 10.1371/journal.pone.0308623 (PMC11309473; doi:10.1371/journal.pone.0308623)
Supplement: S2 Appendix — (DOCX) [file pone.0308623.s002.docx]

## MUSCULOSKELETAL HEALTH QUESTIONNAIRE (MSK-HQ)

This questionnaire is about your **joint, back, neck, bone and muscle symptoms** such as aches, pains and/or stiffness.

Please focus on the particular health problem(s) for which you sought treatment from this service.

*For each question* ***tick*** *(****🗸****)* ***one*** ***box*** *to indicate
which statement best describes you* ***over the last 2 weeks****.*

| 1. Pain/stiffness during the day How severe was your usual joint or muscle pain and/or stiffness overall during the day in the last 2 weeks? | Not at all | Slightly | Moderately | Fairly severe | Very severe |
| --- | --- | --- | --- | --- | --- |
|  | □ 4 | □ 3 | □ 2 | □ 1 | □ 0 |
| 2. Pain/stiffness during the night How severe was your usual joint or muscle pain and/or stiffness overall during the night in the last 2 weeks? | Not at all | Slightly | Moderately | Fairly severe | Very severe |
|  | □ 4 | □ 3 | □ 2 | □ 1 | □ 0 |
| 3. Walking How much have your symptoms interfered with your ability to walk in the last 2 weeks? | Not at all | Slightly | Moderately | Severely | Unable  to walk |
|  | □ 4 | □ 3 | □ 2 | □ 1 | □ 0 |
| 4. Washing/Dressing How much have your symptoms interfered with your ability to wash or dress yourself in the last 2 weeks? | Not at all | Slightly | Moderately | Severely | Unable to wash or dress myself |
|  | □ 4 | □ 3 | □ 2 | □ 1 | □ 0 |
| 5. Physical activity levels How much has it been a problem for you to do physical activities (e.g. going for a walk or jogging) to the level you want because of your joint or muscle symptoms in the last 2 weeks? | Not at all | Slightly | Moderately | Very much | Unable  to do physical activities |
|  | □ 4 | □ 3 | □ 2 | □ 1 | □ 0 |
| 6. Work/daily routine  How much have your joint or muscle symptoms interfered with your work or daily routine in the last 2 weeks (including work & jobs around the house)? | Not at all | Slightly | Moderately | Severely | Extremely |
|  | □ 4 | □ 3 | □ 2 | □ 1 | □ 0 |
| 7. Social activities and hobbies How much have your joint or muscle symptoms interfered with your social activities and hobbies in the last 2 weeks? | Not at all | Slightly | Moderately | Severely | Extremely |
|  | □ 4 | □ 3 | □ 2 | □ 1 | □ 0 |

Please turn the page and continue

| 8. Needing help How often have you needed help from others (including family, friends or carers) because of your joint or muscle symptoms in the last 2 weeks? | Not at all | Rarely | Sometimes | Frequently | All the time |
| --- | --- | --- | --- | --- | --- |
|  | □ 4 | □ 3 | □ 2 | □ 1 | □ 0 |
| 9. Sleep How often have you had trouble with either falling asleep or staying asleep because of your joint or muscle symptoms in the last 2 weeks? | Not at all | Rarely | Sometimes | Frequently | Every night |
|  | □ 4 | □ 3 | □ 2 | □ 1 | □ 0 |
| 10. Fatigue or low energy How much fatigue or low energy have you felt in the last 2 weeks? | Not at all | Slight | Moderate | Severe | Extreme |
|  | □ 4 | □ 3 | □ 2 | □ 1 | □ 0 |
| 11. Emotional well-being How much have you felt anxious or low in your mood because of your joint or muscle symptoms in the last 2 weeks? | Not at all | Slightly | Moderately | Severely | Extremely |
|  | □ 4 | □ 3 | □ 2 | □ 1 | □ 0 |
| 12. Understanding of your condition and any current treatment Thinking about your joint or muscle symptoms, how well do you feel you understand your condition and any current treatment (including your diagnosis and medication)? | Completely | Very well | Moderately | Slightly | Not at all |
|  | □ 4 | □ 3 | □ 2 | □ 1 | □ 0 |
| 13. Confidence in being able to manage your symptoms How confident have you felt in being able to manage your joint or muscle symptoms by yourself in the last 2 weeks (e.g. medication, changing lifestyle)? | Extremely | Very | Moderately | Slightly | Not at all |
|  | □ 4 | □ 3 | □ 2 | □ 1 | □ 0 |
| 14. Overall impact How much have your joint or muscle symptoms bothered you overall in the last 2 weeks? | Not at all | Slightly | Moderately | Very much | Extremely |
|  | □ 4 | □ 3 | □ 2 | □ 1 | □ 0 |

| **Physical activity levels** In the past week, on how many days have you done a total of 30 minutes or more of physical activity, which was enough to raise your heart rate? *This may include sport, exercise and brisk walking or cycling for recreation or to get to and from places, but should not include housework  or physical activity that is part of your job.* | | | | | | | |
| --- | --- | --- | --- | --- | --- | --- | --- |
| None  □ | 1 day  □ | 2 days  □ | 3 days  □ | 4 days  □ | 5 days  □ | 6 days  □ | 7 days  □ |

Thank you for completing this questionnaire.

The MSK-HQ total score is the sum of items 1-14, using the response values provided.
